# Clinical Investigation of Benign Asbestos Pleural Effusion

**DOI:** 10.1155/2015/416179

**Published:** 2015-11-24

**Authors:** Nobukazu Fujimoto, Kenichi Gemba, Keisuke Aoe, Katsuya Kato, Takako Yokoyama, Ikuji Usami, Kazuo Onishi, Keiichi Mizuhashi, Toshikazu Yusa, Takumi Kishimoto

**Affiliations:** ^1^Department of Medical Oncology, Okayama Rosai Hospital, 1-10-25 Chikkomidorimachi, Okayama 7028055, Japan; ^2^Department of Respiratory Medicine, Okayama Rosai Hospital, 1-10-25 Chikkomidorimachi, Okayama 7028055, Japan; ^3^Department of Respiratory Medicine, Chugoku Chuo Hospital, Fukuyama 7200001, Japan; ^4^Department of Medical Oncology, Yamaguchi-Ube Medical Center, 685 Higashikiwa, Ube 7550241, Japan; ^5^Department of Radiology, Okayama University Hospital, 2-5-1 Shikatacho, Okayama 7008558, Japan; ^6^Department of Diagnostic Radiology 2, Kawasaki Medical School, Okayama 7008505, Japan; ^7^Department of Respiratory Medicine, Asahi Rosai Hospital, 61 Hirakochokita, Owariasahi 4880875, Japan; ^8^Department of Respiratory Medicine, Kobe Rosai Hospital, 4-1-23 Kagoikedori, Chuoku, Kobe 6510053, Japan; ^9^Department of Respiratory Medicine, Toyama Rosai Hospital, 992 Rokuromaru, Uozu 9370042, Japan; ^10^Department of Thoracic Surgery, Chiba Rosai Hospital, 2-16 Tatsumidaihigashi, Ichihara 2900003, Japan; ^11^Department of Internal Medicine, Okayama Rosai Hospital, 1-10-25 Chikkomidorimachi, Okayama 7028055, Japan

## Abstract

There is no detailed information about benign asbestos pleural effusion (BAPE). The aim of the study was to clarify the clinical features of BAPE. The criteria of enrolled patients were as follows: (1) history of asbestos exposure; (2) presence of pleural effusion determined by chest X-ray, CT, and thoracentesis; and (3) the absence of other causes of effusion. Clinical information was retrospectively analysed and the radiological images were reviewed. There were 110 BAPE patients between 1991 and 2012. All were males and the median age at diagnosis was 74 years. The median duration of asbestos exposure and period of latency for disease onset of BAPE were 31 and 48 years, respectively. Mean values of hyaluronic acid, adenosine deaminase, and carcinoembryonic antigen in the pleural fluid were 39,840 ng/mL, 23.9 IU/L, and 1.8 ng/mL, respectively. Pleural plaques were detected in 98 cases (89.1%). Asbestosis was present in 6 (5.5%) cases, rounded atelectasis was detected in 41 (37.3%) cases, and diffuse pleural thickening (DPT) was detected in 30 (27.3%) cases. One case developed lung cancer (LC) before and after BAPE. None of the cases developed malignant pleural mesothelioma (MPM) during the follow-up.

## 1. Introduction

Asbestos-related pathological changes of the pleura include pleural plaques, malignant pleural mesothelioma (MPM), diffuse pleural thickening (DPT), and benign asbestos pleural effusion (BAPE). BAPE is a nonmalignant pleural disease initially described in 1964 [1]. It is also termed asbestos pleuritis. Once a patient is diagnosed with BAPE, he or she is compensated by workers' compensation in Japan. Epler et al. [2] advocated diagnostic criteria for BAPE, which include (1) previous asbestos exposure, (2) determination of pleural effusion by chest X-ray or thoracentesis, and (3) the absence of other causes of effusion. They also stated that follow-up assessments for at least 3 years were essential to confirm the diagnosis and to exclude the development of malignant diseases such as MPM or lung carcinomatous pleuritis. Later, Hillerdal and Ozesmi [3] described that a 1-year follow-up would be sufficient based on a detailed exploration including computed tomographic (CT) scanning. Most of the previous studies included small numbers of patients and were undertaken in the 1980s, so no detailed information is available about the disease.

In the current study, we retrospectively analysed the clinical features of BAPE in patients in Japan. The aim of the study was to clarify the clinical features of BAPE and to suggest more practical diagnostic standard for the disease.

## 2. Patients and Methods

### 2.1. Subjects

Enrolled patients were referred to Rosai Hospital and affiliated hospitals in Japan for an examination for pleural effusion and were finally diagnosed with BAPE. The criteria of enrolled patients were as follows: (1) previous history of asbestos exposure obtained by an in-person questionnaire or interview; (2) presence of pleural effusion determined by chest X-ray, CT, and thoracentesis; and (3) the absence of other causes of effusion. The pleural fluid was collected by thoracentesis or thoracoscopy, and information on cell classification, cytological analysis, and the biochemical examination was extracted from the medical records. Hyaluronic acid (HA), adenosine deaminase (ADA), and carcinoembryonic antigen (CEA) were included among the clinical laboratory tests. The HA concentration was determined using a latex agglutination turbidimetric immunoassay. ADA was measured using an enzymatic technique. CEA was measured using a chemiluminescent immunoassay.

### 2.2. Data Collection and Analysis

Clinical and demographic information was obtained from the medical records at each facility. The information included age, gender, smoking status, initial symptoms, and results of laboratory testing of the pleural effusion. The work histories, those of the family members, and residential histories were investigated to assess the patient's history of asbestos exposure.

The radiological images were sent to Okayama Rosai Hospital for review. Characteristic radiological findings associated with asbestos exposure were assessed as the presence of pleural effusion, asbestosis, rounded atelectasis, pleural plaques, and DPT. Asbestosis was classified on chest X-rays according to perfusion rate (PR) based on the International Labour Organization (ILO) criteria [4]. DPT was defined as pleural thickening of more than 5 mm on chest X-rays, extending for more than half of the lateral thoracic wall (LTW) in cases of unilateral DPT or more than quarter of the LTW in cases of bilateral DPT [5]. The presence of pleural effusion, rounded atelectasis, and pleural plaques was assessed on chest CT.

Survival data were determined from the day pleural effusion was detected to the day of death or last follow-up and analysed using the Kaplan-Meier method with SPSS 11.0 software (SPSS, Inc., Chicago, IL, USA).

This study was done according to the Ethical Guidelines for Epidemiological Research by the Japanese Ministry of Education, Culture, Sports, Science, and Technology and the Ministry of Health, Labour, and Welfare. This study was approved by Japan Labour Health and Welfare Organization and the institutional review boards of each institution. Patient confidentiality was strictly maintained. This study was carried out according to the principles set out in the Declaration of Helsinki.

## 3. Results

### 3.1. Patient Characteristics

One hundred ten patients from 9 institutions fulfilled the enrolled criteria based on the descriptions in their medical records and review of the radiographs between 1991 and 2012. Characteristics of the patients are shown in Table 1. Smoking history was obtained in 63 cases including 56 ever/current smokers and 7 never smokers, with the median (range) pack-years of 34.5 (0–112). Pleural effusion was found in 56 cases in the right, 25 in the left, and 27 in both thoracis. Sixty-five patients visited the clinic for subjective symptoms, and pleural effusion was detected at the regular medical check-up in 35 cases without any symptoms. Pleural effusion was detected during the treatment of other diseases in another 15 cases. Thoracentesis was performed in all patients to collect pleural fluid. Thoracoscopic exploration was done in 78 patients to exclude carcinomatous pleuritis or MPM and to confirm the diagnosis of BAPE.

### 3.2. Asbestos Exposure History

A history of asbestos exposure was reported by 109 patients, with one patient whose detailed information of asbestos exposure was not obtained. Among the 109 patients, 108 patients had a history of occupational asbestos exposure and one patient had a history of environmental asbestos exposure. The occupational categories associated with asbestos exposure are shown in Table 2. The median (range) age of the first exposure to asbestos was 21.5 (14–58) years. The median (range) duration of asbestos exposure was 31 (0.75–50) years and the median (range) period of latency for disease onset of BAPE was 48 (17–76) years.

### 3.3. Characteristics of the Pleural Effusion

Information regarding the pleural effusion was obtained in 104 cases. The gross impression of the pleural fluid was bloody in 75 cases, light yellow in 27, and light brown and dark red in 1 case each. The effusions were exudative in all cases. A cellular classification of the fluid was obtained in 57 cases and the median proportions of lymphocytes, macrophages, neutrophils, and eosinophils were 77.7%, 9.7%, 8.0%, and 8.0%, respectively. The HA concentration was determined in 106 cases and the mean (standard deviation) concentration was 39,840 (40,228) ng/mL. Mean (standard deviation) values of ADA and CEA were 23.9 (24.9) IU/L and 1.8 (1.3) ng/mL, respectively.

### 3.4. Concomitant Asbestos-Related Findings

As shown in Table 3, pleural plaques were detected in 98 cases (89.1%), among which 76 cases were calcified. Asbestosis was present in 6 cases, rounded atelectasis was detected in 41 cases (37.3%), and DPT was detected in 30 cases (27.3%). One of the cases developed lung cancer (LC) before and after diagnosis of BAPE. The patient had undergone right upper lobectomy for LC two years before his BAPE diagnosis and left partial lobectomy for another LC two years after his BAPE diagnosis.

### 3.5. Clinical Course

**Figure 1:**
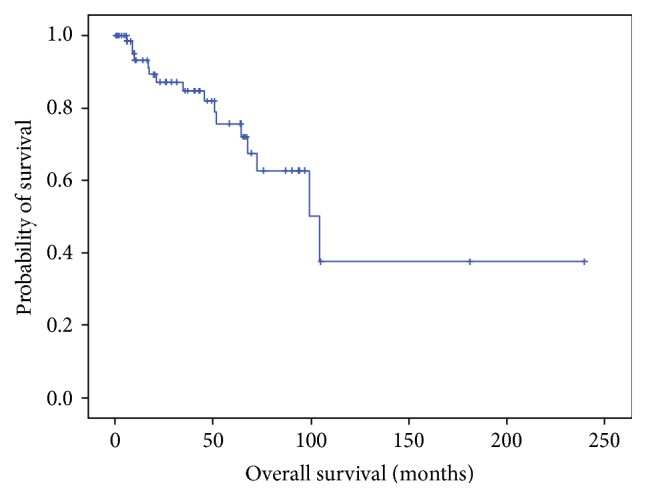
Overall survival of patients with benign asbestos-related pleural effusions at Okayama Rosai Hospital.

In most of the cases, thoracentesis and/or thoracotomy were done to collect the fluid and drain the pleural effusion. Oral steroids were prescribed in 5 cases and one of them demonstrated temporal decrease of the effusion. Survival data was obtained in 70 cases from Okayama Rosai Hospital. As shown in Figure 1, median overall survival was 104.2 months (95% confidence interval (CI), 67.3–141.0 months) after a median observation period of 73.0 months (95% CI, 16.2–268.2 months). There were 17 dead cases out of 70 cases at the analysis. The causes of death were determined in 11 cases including 7 respiratory failure cases and each 1 of renal failure, suicide, septic shock due to urinary tract infection, and death of old age. There were 9 cases that developed DPT out of the 17 cases, including the 7 dead cases of respiratory failure. At the time of the analysis, none of the cases had developed MPM.

## 4. Discussion

In the current study, we examined the clinical features of BAPE and demonstrated that BAPE developed after long-term asbestos exposure. In a previous report, BAPE occurred 15–20 years after exposure and was more common in younger patients aged 21–40 years [6]. In another report, the interval between asbestos exposure and presentation of BAPE varied between 5 and more than 30 years, and early onset was correlated with higher asbestos exposure [7]. Wagner reported that BAPEs were usually unilateral, and the most common manifestation of asbestos-related pleural disease occurred 10 to 20 years after exposure [8]. A limitation of these earlier studies is that the diagnosis criteria of BAPE were ambiguous in the studies. The median latency period between asbestos exposure and BAPE development in the current study was 48 years, which was similar to that observed for MPM (41 years), LC (47 years), and asbestos-induced DPT (46 years) in our previous reports [4, 9, 10]. We consider that BAPE develops after a long latency period in those with a history of asbestos exposure. There is one point, however, that most of the patients of BAPE in the current study have associated with other asbestos-related lesions such as rounded atelectasis and/or diffuse pleural thickening. It is possible that BAPE might have been developed earlier in these cases, and this could be an explanation of the longer latency of BAPE than previously published. The current study suggests that BAPE can develop after moderate-to-high levels of exposure to asbestos, because the occupational category of the subjects in the current study included those of relatively high levels of asbestos exposure such as asbestos product manufacturing, construction, and shipbuilding, although the correlation between the exposure amount and development of BAPE is unclear. The subjects in the current study included substantial portion of those with smoking history. To our knowledge, the correlation between BAPE and smoking history has not been reported.

The diagnosis of BAPE should be based on a history of asbestos exposure and an exclusion of other causes of effusion such as tuberculous pleuritis, bacterial pleuritis, collagen diseases, heart failure, and malignant conditions such as MPM and LC. In our analysis, the gross impression of the pleural fluid was bloody in 72% of the cases, and cellular classification of the fluid demonstrated lymphocyte dominancy. These results are similar to those of a previous report showing that the effusion was exudative and could be hemorrhagic, as well as predominantly eosinophilic [11].

In cases of LC, tumor cells are detected in the fluid in more than 60% of cases [12]. In cases with MPM, tumor cells can be detected in the pleural fluid, but the detection rate has been reported as less than 30% [13]. Tuberculosis pleuritis or bacterial pleuritis could be diagnosed by staining for acid-fast bacteria, polymerase chain reaction detection, or bacterial culture, although the detection rate is usually low. These analyses may not always determine the diagnosis but should be undergone to exclude MPM, LC, and tuberculosis or bacterial pleuritis and to make the diagnosis of BAPE.

In addition, we analysed some markers such as HA concentration, ADA, and CEA. Recently, we reported the clinical usefulness of HA for the differential diagnosis of MPM and BAPE [14]. In cases with tuberculosis pleuritis, elevated values of ADA could help in the diagnosis [15]. However, elevated ADA may not be limited to tuberculous pleuritis, as it is also present in LC or MPM [16]. In cases with elevated CEA values, carcinomatous pleuritis is strongly suggested [17]. These markers should be determined to exclude these conditions and to confirm a diagnosis of BAPE. However, the differential diagnosis of MPM and BAPE is especially difficult, even when based on these markers. Especially in cases with exudative pleural effusions, thoracoscopic exploration and pleural biopsy should be performed to exclude MPM and confirm the diagnosis of BAPE [18].

Based on the findings in the current study and previous reports, we propose more practical diagnostic standard for the diagnosis of BAPE including (1) asbestos exposure history, (2) exudative effusion, and (3) exclusion of other pleuritides such as LC, MPM, and tuberculous pleuritis by radiological examination and pleural biopsy via thoracoscopy. Additional diagnostic information is as follows: (1) in cases thoracoscopy could not be undergone, the diagnosis should be discussed based on the bacteriological examination and biochemical markers such as CEA, ADA, and HA; in cases with elevated CEA (>5 ng/mL), ADA (>35 IU/L), or HA (>100,000 ng/dL), carcinomatous pleuritis, tuberculous pleuritis, or MPM is more likely, respectively; and (2) in cases with some concomitant medical problem such as autoimmune diseases, the activity of the disease should be carefully evaluated, because autoimmune diseases such as systemic lupus erythematosus or rheumatoid arthritis could involve the pleura and cause pleural effusion (Table 4).

“Benign” is meant to refer to a nonmalignant process, but these effusions can be associated with significant morbidity [19]. The effusion generally takes a long time to resolve. It may resolve spontaneously or be followed by DPT, which causes extrapulmonary restriction and may thereby ultimately become disabling. Previous studies reported that a considerable number of patients with BAPE subsequently developed DPT [2, 3]. Actually, in our previous study, half the patients with asbestos-induced DPT had a history of BAPE [4]. Furthermore, in the current study one patient developed LC before and after being diagnosed with BAPE. The risks of developing MPM or LC in patients with BAPE are increased compared with those of the general population because of their past history of asbestos exposure. Particular attention should be paid to the management of patients with BAPE.

There are a few limitations to the current study. First, this was a retrospective study. Second, pathological analyses including immunohistochemistry were not reviewed. In addition, there are recent reports that increased uptake of fluorodeoxyglucose (FDG) by positron emission tomography (PET) may be a useful marker to distinguish MPM from benign pleural disease [20, 21]. In addition, recent reports revealed that biomarkers such as soluble mesothelin-related peptides (SMRP) are selectively elevated in patients with MPM [22, 23]. A clinical study to evaluate the utility of PET and/or SMRP for the differentiation between MPM and BAPE is warranted.

## 5. Conclusions

BAPE develops after a long latency period after past asbestos exposure. The diagnosis of BAPE should be based on the exclusion of other pleural diseases. A thorough evaluation, including diagnostic thoracentesis and cytological and bacterial analysis, must be performed. Clinical markers such as HA, ADA, and CEA might help with the differential diagnosis. However, thoracoscopic exploration and pleural biopsy should be performed to confirm a diagnosis of BAPE.

## Figures and Tables

**Table 1:** Patient characteristics.

Age (*n* = 110)	
Median (range)	74 (36–90)
Gender (*n* = 110)	
Male/female	110/0
Smoking history (*n* = 63)	
Ever/current	56
Never	7
Symptoms (*n* = 65, multiple answers)	
Dyspnea	34
Cough	15
Chest pain	13
Fever	3
Palpitation	2
Sputum	1
Wheezing	1
Back pain	1
Weight loss	1
Fatigue	1

**Table 2:** Occupational category related to asbestos exposure.

Shipbuilding	25
Construction	20
Chemical facility	10
Asbestos products manufacturing	8
Electrical work	8
Plumbing	7
Asbestos transportation	5
Moisturizing work	4
Asbestos spraying	3
Steel production	3
Demolition work	2
Automobile manufacturing	2
Heat insulation	2
Firebrick manufacturing	2
Glasswork	1
Metallic product manufacture	1
Furnace installation	1
Coating industry	1
Shipman	1
Others	2

Total	108

**Table 3:** Concomitant asbestos-related radiological findings.

Findings		*n*	%
Pleural plaques		98	89.1
Calcified		76	
Asbestosis		6	5.7
PR^†^	1	3	
	2	2	
	3	1	
Rounded atelectasis		41	37.3
DPT^‡^		30	27.3

^†^Perfusion rate, ^‡^diffuse pleural thickening.

**Table 4:** Proposed diagnostic criteria of benign asbestos pleural effusion.

Diagnostic criteria	

(1) Asbestos exposure history.	
(2) Exudative effusion.	
(3) Exclusion of other pleuritides such as lung cancer, MPM^†^, and tuberculous pleuritis by radiological examination and pleural biopsy via thoracoscopy.	

Additional diagnostic information	

(1) In cases thoracoscopy could not be undergone, the diagnosis should be discussed based on the bacteriological examination and biochemical markers below.	
(a) Elevated carcinoembryonic antigen (>5 ng/mL) suggests carcinomatous pleuritis.	
(b) Elevated adenosine deaminase (>35 IU/L) suggests tuberculous pleuritis.	
(c) Elevated hyaluronic acid (>100,000 ng/dL) suggests MPM.	
(2) In cases with some concomitant medical problem such as autoimmune diseases, the activity of the disease should be carefully evaluated.	

^†^Malignant pleural mesothelioma.
